# COSMIC 2005

**DOI:** 10.1038/sj.bjc.6602928

**Published:** 2006-01-17

**Authors:** S Forbes, J Clements, E Dawson, S Bamford, T Webb, A Dogan, A Flanagan, J Teague, R Wooster, P A Futreal, M R Stratton

**Affiliations:** 1 Wellcome Trust Sanger Institute, Wellcome Trust Genome Campus, Hinxton, Cambridgeshire, CB10 1SA, UK; 2 Mayo Clinic, Department of Laboratory Medicine and Pathology, 200 First Street SW, Rochester, MN 55905, USA; 3 The Institute of Orthopaedics, UCL, Stanmore, Middlesex, HA7 4LP, UK

**Keywords:** somatic, mutation, database, website

## Abstract

The Catalogue Of Somatic Mutations In Cancer (COSMIC) database and web site was developed to preserve somatic mutation data and share it with the community. Over the past 25 years, approximately 350 cancer genes have been identified, of which 311 are somatically mutated. COSMIC has been expanded and now holds data previously reported in the scientific literature for 28 known cancer genes. In addition, there is data from the systematic sequencing of 518 protein kinase genes. The total gene count in COSMIC stands at 538; 25 have a mutation frequency above 5% in one or more tumour type, no mutations were found in 333 genes and 180 are rarely mutated with frequencies <5% in any tumour set. The COSMIC web site has been expanded to give more views and summaries of the data and provide faster query routes and downloads. In addition, there is a new section describing mutations found through a screen of known cancer genes in 728 cancer cell lines including the NCI-60 set of cancer cell lines.

All cancers arise through the acquisition of a number of DNA sequence mutations, some of which confer growth advantage and drive the clonal expansion of the tumour cells (Vogelstein and Kinzler, 1998). At the DNA sequence level the mutations include base substitutions, deletions, amplifications and rearrangements. It is likely that many somatic mutations are a consequence of defects in DNA repair and maintenance (Slupphaug *et al*, 2003; Barnes and Lindahl, 2005) or past exposure to mutagens (Luch, 2005) or both of these phenomena. Are all somatic mutations critical for the development of the tumour in which they are found? Probably not, but the proportion of mutations that are causally implicated in cancer is unclear and certainly varies from tumour to tumour (Wang *et al*, 2004; Davies *et al*, 2005; Stephens *et al*, 2005; Bignell *et al*, 2005). Differentiating passenger events from disease causing mutations is a challenge, particularly for genes that are infrequently mutated or have silent or noncoding mutations. This contrasts with genes that are frequently mutated, beyond what would be expected by chance, or have mutations that cluster in key amino-acid residues or functional protein domains. In these cases the genetic evidence on its own strongly implies these genes are involved in the development of cancer. What is clear is the utility of mutation data.

The small intragenic mutation data that defines known cancer genes is buried in the scientific literature. There are extensive databases and web sites that actively curate the literature for germline mutations in cancer genes, for example HGVbase (Fredman *et al*, 2002) and the Human Gene Mutation Database (HGMD, Stenson *et al*, 2003). In addition, there are many databases that store and serve somatic mutation data for single genes (see http://www.hgvs.org for an extensive list). Some of these are actively maintained, such as those for TP53 (Olivier *et al*, 2002; Béroud and Soussi, 2003), however, most are not updated. Furthermore, there is wide variation in the data that is stored, the extent of queries that can be levelled at the data and the ability to display and download the results. Although all these resources have value they are dispersed across the internet and thus it is difficult to make direct comparisons between cancer genes.

Since the early days of sequencing genes in tumours there have been reports of infrequently mutated genes and occasionally genes that appear to have no mutations. This data is now joined by the results of the systematic sequencing of genes in tumours (Bardelli *et al*, 2003; Wang *et al*, 2004; Davies *et al*, 2005; Bignell *et al*, 2005; Stephens *et al*, 2005) that also report infrequently mutated genes and many more genes with no mutations. Is this data worth preserving? Definitely yes, both to disseminate the mutation data to a wide audience and as a means of preserving the negative data.

The Catalogue of Somatic Mutations in Cancer, COSMIC, (http://www.sanger.ac.uk/cosmic) was launched in 2004 as a free resource to hold and display somatic mutation data for four genes; BRAF, HRAS, KRAS and NRAS (Bamford *et al*, 2004). The data in COSMIC has expanded to include data on 538 genes, 124 367 tumours with 23 157 mutations. The web site has been expanded to provide summary pages for the genes, tissue types, references, samples and mutations. In addition, there are new sections detailing the results of our sequencing of known cancer genes in 728 publicly available cancer cell lines that incorporate the NCI-60 cancer cell lines including loss of heterozygosity data and copy number information for many of these cancer cell lines.

## DATA CURATION

The genes that have been selected for curation are a subset from the Cancer Gene Census (http://www.sanger.ac.uk/CGP/Census Futreal *et al*, 2004) and other genes that have been screened for somatic mutations with either negative or inconclusive results. The data held in COSMIC is extracted from the literature as described in Bamford *et al*, 2004. Once a gene is included in COSMIC there is an ongoing process to curate additional data after it is published. There is usually a delay between publication of data and its appearance in COSMIC while the data is curated.

To enhance the utility of COSMIC we standardise the curated data. We extract the tissue and histology for each sample and map the definitions to the COSMIC classification tables (see http://www.sanger.ac.uk/genetics/CGP/cosmic/data/cosmic_classification_alias_list_01_11_05.xls). This yields a standard set of tumour descriptions that can be queried through the web site. The original definition is always maintained in the database. In a similar fashion, a single DNA sequence is held for each transcript. The transcript sequence is translated to give the protein sequence used by COSMIC. This information is available for each gene and all mutations are mapped to these standard sequences. For example, all BRAF V599E mutations are remapped to amino acid 600 in the COSMIC BRAF protein sequence (see Davison *et al*, 2005 for a typical example).

### Potential data biases

The data held in COSMIC that is extracted from the literature is likely to have a number of biases. There is potential for publication prejudice where positive data is more likely to appear in print than negative data. There are almost certainly biases in the samples that have been analysed as many studies are performed using tumours from Europe and the USA. Where particular patient groups appear interesting there is often a surge of analysis that can distort the mutation landscape, for instance the reported population bias in EGFR mutations (Paez *et al*, 2004). Furthermore, it is a common practice to screen mutation hotspots in known cancer genes, for example, the selective analysis of codons 12 and 13 in the RAS genes. Where possible all data is entered in to COSMIC rather than selecting specific data sets. When viewing the data in COSMIC there is always a link to the publications that were curated making it possible to view the original data, samples and methods to understand any biases.

## DATABASE

The COSMIC database is implemented in Oracle. The schema has expanded since the launch of COSMIC to encompass additional details and enhance the tracking of the curation process (see Supplementary data).

The main development of the database has been the introduction of feature tables that are linked to the individual and tumour tables. The feature tables are a generic approach to storing any information relating to the individual and tumour. The features are grouped into feature types, for example, ethnicity. Any ethnic name can be added to this feature type. A more complex feature type is cigarette smoking history. The values that have been stored so far for this feature type include values expressed as pack years as well as less specific comments, such as smoker, nonsmoker, ex-smoker and never-smoker. This system allows COSMIC to capture the wide range of information reported in the literature. It also accepts different data content for different genes, for example, drug response information for tumours with and without EGFR mutations. The other noteworthy addition to the COSMIC schema is a pair of tables that store external data sources for the samples held in COSMIC (see Other Data Types below).

## WEB SITE

The COSMIC web site has been further developed to provide faster access to the data, new views and summaries and new links to aid navigation around the various pages.

**Figure 1:**
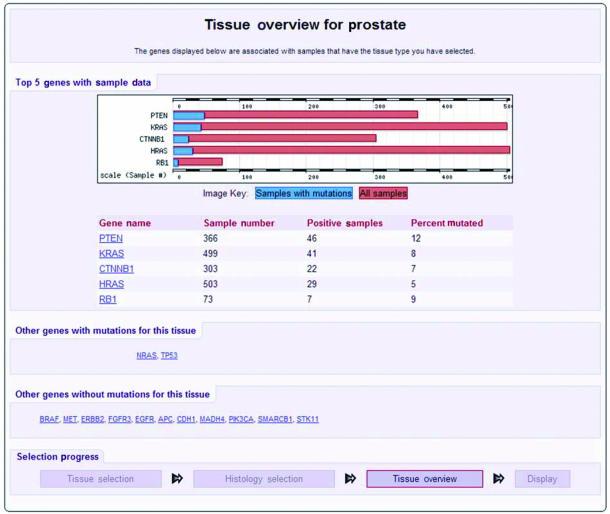
Tissue overview. The mutation data for a selected tissue is presented in a summary format, in this case for prostate. The top 5 genes with data in COSMIC are selected as the genes with the highest rank score using the method; RankScore=number of mutations/number of samples – 1.6449 × squareroot((number of mutations/number of samples) × (1–(number of mutations/number of samples)/number of samples). The data is presented in both graphical and tabular formats. Further genes with and without mutations for the selected tissue are listed. All of the gene names can be followed to view the details of the mutations.

There are two routes to the data; selecting a gene or a tissue. The gene selection is either alphabetical or by chromosome position. There are two tissue selection paths. The Browse by Tissue route presents a list of tissues, subtissues then histologies and subhistologies, which culminate in a tissue overview display (Figure 1). The Quick Tissue path proceeds straight from the tissue selection to the overview page.

**Figure 2:**
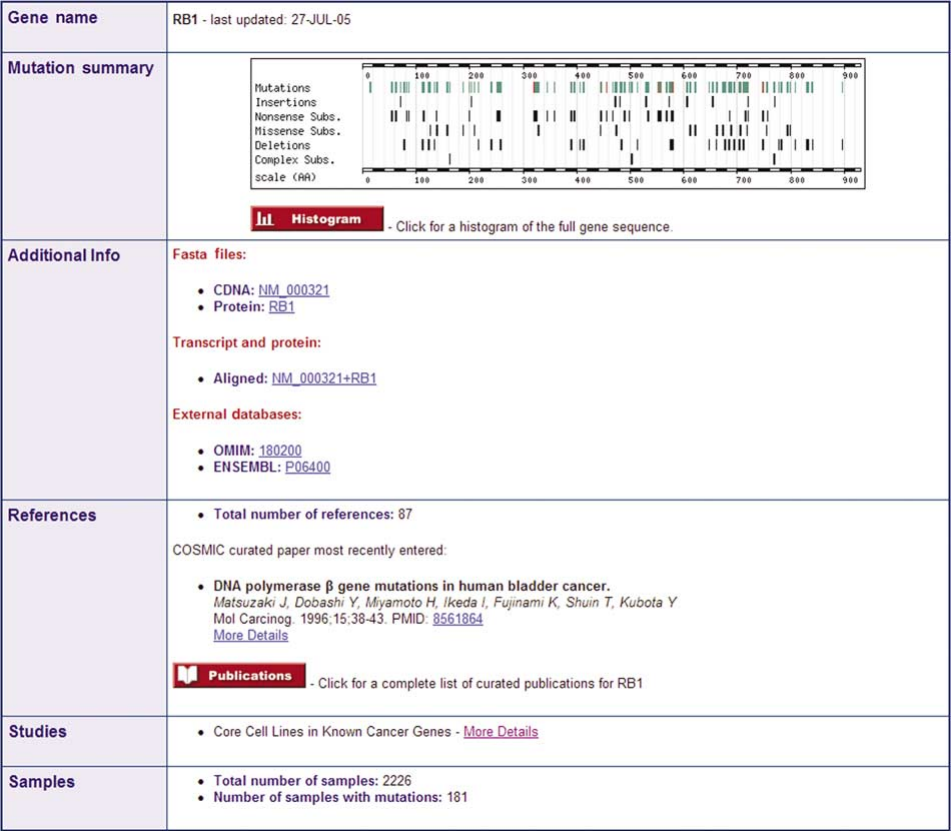
Gene summary. The initial output for a gene is a graphical view of the mutations distributed along the linear amino acid sequence of the gene. This is the data for RB1. The positions of the mutations are shown by tick marks with tracks showing the total number of mutations and mutations that are insertions, nonsense substitutions, missense substitutions, deletions and complex substitutions. In addition the summary presents the number of references curated, the number of samples for the gene and the number of samples with mutations. There are multiple links from this view leading to web pages describing more details of the mutations, the gene and the references that have been curated.

The gene summary page provides an overview of the data for each gene (Figure 2). The position of recorded mutations is shown on an overview of the protein sequence with links to the gene histogram page. In addition the gene summary has links to external data sources for the gene, the references that have been curated and an overall sample and mutation count. The gene histogram page has been developed from the original web site to show the mutations either on the protein or cDNA sequence but still shows the mutation position, frequency data by tumour type and details of the mutations (not shown). The gene histogram display now also maps the positions of insertions, deletions and complex mutations. The reference summary page presents a list of the genes that were screened in each paper, the samples that had mutations with details of the mutation and the names of the samples that had no mutations in the genes that were screened (not shown). The details for each of the samples and each mutation are presented in two separate summary pages (not shown).

## MUTATION CONTENT

The genes in COSMIC can be split into three categories. In all, 28 genes in COSMIC are considered as causal cancer genes in the Cancer Gene Census where the genetic and biological data (where available) indicates that mutations in the genes are almost certainly involved in the development of cancer (Table 1). Of these, 25 have a mutation frequency above 5% in one or more tumour type while the other three, ERBB2, FGFR2 and SUFU, have biologically plausible mutations but a low mutation frequency (mutation frequencies in all available data are; 1.2% for ERBB2, 2% for FGFR2 and 1.6% for SUFU). On the COSMIC web site these genes are grouped in the gene selection page. The data is current for all of the genes except TP53. The results for TP53 are essentially additional information from other work. They have been included in COSMIC but do not constitute a comprehensive survey of TP53 mutation data. Other resources such as the IARC TP53 database (Olivier *et al*, 2002) give a far more extensive set of TP53 data.

The second set of genes in COSMIC have somatic mutations in cancers, however the frequency of mutations is low, generally <5% in all tumour types, and/or they are not located in known functionally significant positions in the proteins. This set comprises 180 genes. The majority of these genes have been screened in a small number of samples. However, a small subset, for example, ACVR1B and CSF1R, have been screened in many cancers. The role of these mutated genes in the development of cancer is unclear and the mutations could be termed ‘somatic variants of unknown significance’. In all likelihood most are not causally implicated in oncogenesis, that is, the mutations are passenger (also known as bystander) mutations. However, it is equally plausible that a minority is involved in cancer development, although it is currently not possible to determine which.

The final set of genes has been screened for mutations but none have been reported. This set of genes is large (333) with the data coming from the sequencing of all 518 protein kinase genes in: 25 breast cancers (Stephens *et al*, 2005), 33 lung cancers (Davies *et al*, 2005) and 13 testicular germ cell tumours (Bignell *et al*, 2005). In general, this type of data is either not present in the literature or the description is cursory making it difficult to enter in COSMIC. If mutations are found in these genes in the future, the status of the genes in COSMIC would be modified.

## CANCER CELL LINES AND KNOWN CANCER GENES

Cancer cell lines have been used extensively in the biological characterisation of cancer and in the analysis of both novel and routinely used anticancer drugs. On the whole this has taken place with little or no consideration of the DNA sequence of known cancer genes in these samples. To redress this imbalance COSMIC now displays mutation data that we have generated from known cancer genes in the NCI-60 cell line panel of 59 lines and a further 669 cancer cell lines (http://www.sanger.ac.uk/genetics/CGP/CellLines/). Some of these cell lines have been sequenced in the past. For example, TP53 has been sequenced in the NCI-60 (O’Connor *et al*, 1997) while other lines have been used as positive controls in mutation screening experiments. Rather than curate this rather piecemeal set of results, we have begun to systematically resequence known cancer genes in this group of cell lines.

## OTHER DATA TYPES

There is additional genetic data for the samples being analysed by us (http://www.sanger.ac.uk/genetics/CGP). In all, 829 cancer cell lines in COSMIC have loss of heterozygosity maps produced by genotyping 395 polymorphic CA repeats from across the genome. The samples analysed in the protein kinase mutation screen (http://www.sanger.ac.uk/genetics/CGP/Kinases) and normal samples from the same individuals have been genotyped with the Affymetrix 10k SNP array. This data has also been used to calculate loss of heterozygosity maps. In addition, the intensity data from the SNP arrays has been used to generate chromosome copy number maps. The SNP and CA repeat data is integrated with the mutation data to provide a wider genetic perspective of these samples.

## FUTURE DIRECTIONS

The publication of data from systematic mutation screens provides a new avenue for COSMIC. The volume of systematic data is likely to grow and provide a wider insight into the mutation burden in cancer. The screening of known cancer genes in cancer cell lines provides a resource to both the genetics community and those interested in the biology of these cell lines. We intend to expand this data further.

The value of small intragenic mutation data can be enhanced by integrating other data types. As a first step, we have integrated genotyping and copy number data. In the future, we hope to incorporate other somatic mutation data to further expand the content of COSMIC. In the meantime, there are plans for the continued curation of the cancer mutation literature to expand the number of known cancer genes.

## Figures and Tables

**Table 1:** Mutation statistics for the known cancer genes curated in COSMIC

**Gene**	**References**	**Unique mutations**	**Samples with mutations**	**Samples without mutations**
ABL1	18	52	172	552
BRAF	144	77	2767	11509
CEBPA	1	10	10	127
CTNNB1	240	261	1466	10643
EGFR	39	139	685	5398
ERBB2	8	12	20	1693
FGFR2	5	6	5	237
FGFR3	29	21	484	1507
FLT3	50	46	1493	5859
GATA1	4	10	15	69
HRAS	251	28	472	11462
JAK2	9	1	473	568
KIT	113	247	768	2421
KRAS	749	60	8402	29328
MET	29	29	66	1503
MSH6	11	18	89	588
NOTCH1	1	64	72	48
NRAS	313	33	1110	13378
PDGFRA	17	35	207	1060
PIK3CA	9	62	310	1988
PTEN	180	678	1243	7830
PTPN11	9	43	110	2268
RB1	59	126	168	1330
RET	48	35	218	1097
SMARCB1	21	78	193	1348
SMO	7	17	25	234
SUFU	3	4	4	240
TP53	3	9	10	61
				
Totals	2370	2201	21 057	114 346

The data for TP53 is not a comprehensive review of the literature for this gene. Some of the samples screened for mutations in other genes were incidentally screened through TP53 and this data has been captured.
