# Supplementary figures and images for: COSMIC 2005
Source: Br J Cancer. 2006 Jan 17;94(2):318–22. doi: 10.1038/sj.bjc.6602928 (PMC2361125; doi:10.1038/sj.bjc.6602928)

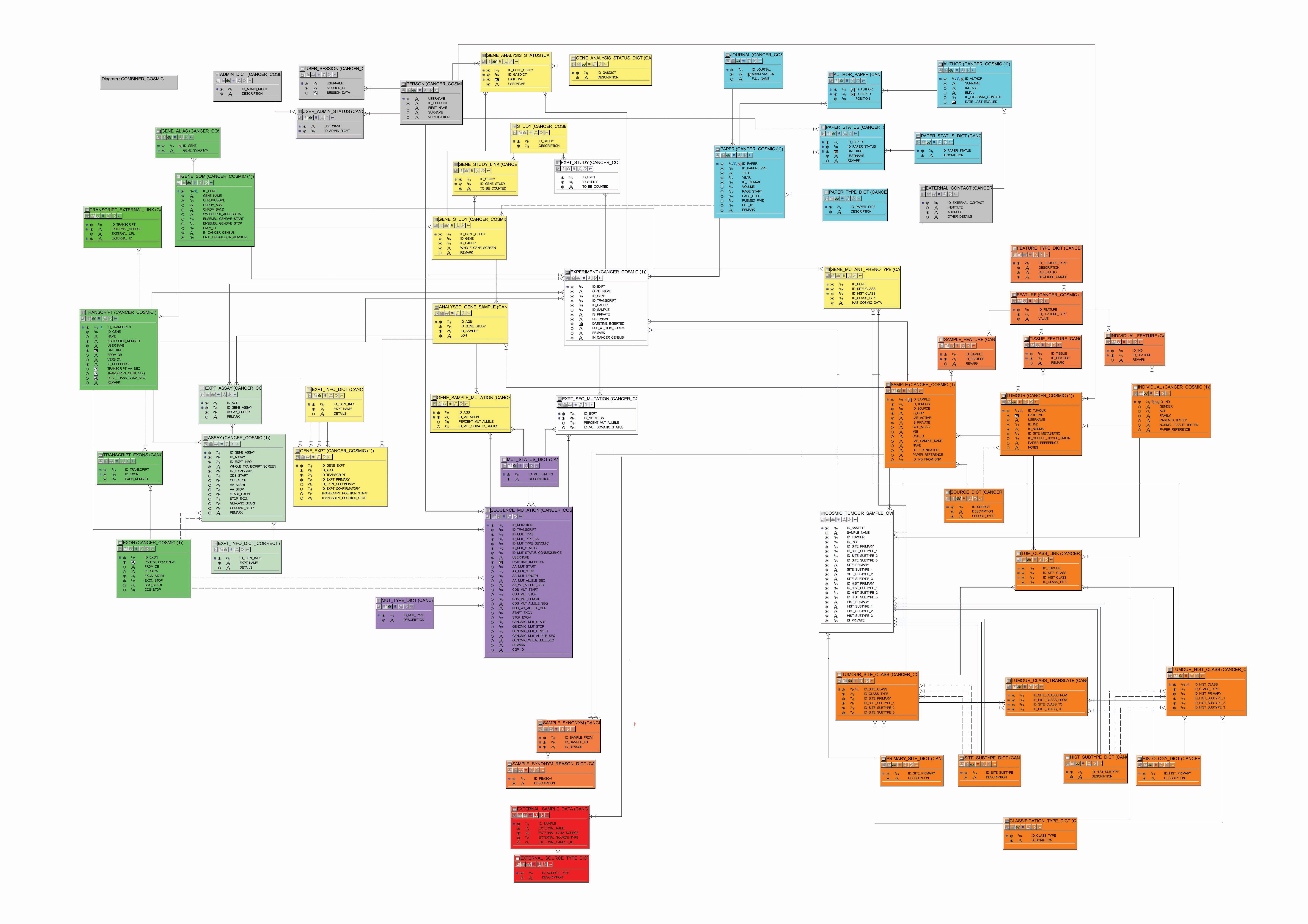

Supplement: supplementary data [file 94-6602928x1.jpg]
